# The impact of aging and atherosclerotic risk factors on transthoracic coronary flow reserve in subjects with normal coronary angiography

**DOI:** 10.1186/1476-7120-10-20

**Published:** 2012-05-14

**Authors:** Maurizio Galderisi, Fausto Rigo, Sonia Gherardi, Lauro Cortigiani, Ciro Santoro, Rosa Sicari, Eugenio Picano

**Affiliations:** 1 Division of Cardioangiology, Department of Clinical and Experimental Medicine, Federico II University Hospital, Naples, Italy; 2 Division of Cardiology, “Dell’Angelo” Hospital, Mestre, Italy; 3 Division of Cardiology, Bufalini Hospital, Cesena, Italy; 4 Division of Cardiology, Lucca Hospital, Lucca, Italy; 5 Institute of Clinical Physiology, National Council of Research, Pisa, Italy; 6 Department of Clinical and Experimental Medicine, Echo Lab, Cardioangiology Unit, Edificio 1, Federico II University Hospital, Via S. Pansini 5, Naples, 80131, Italy

**Keywords:** Coronary flow reserve, Aging, Atherosclerotic risk factors, Transthoracic Doppler echocardiography, Stress echocardiography.

## Abstract

Age may affect coronary flow reserve (CFR) especially in subjects with atherosclerotic risk factors (ARFs). The aim of this prospective, multicenter, observational study was to determine the effects of aging on CFR in patients with normal epicardial coronary arteries and ARFs. Three-hundred-thirty-five subjects (mean age = 61 years) with at least one ARF but normal coronary angiography underwent high-dose dipyridamole stress-echo with Doppler evaluation of left anterior descending artery. CFR was calculated as the ratio between hyperemic and resting coronary diastolic peak velocities. Patients were divided in age quartiles. CFR was progressively reduced with aging (1^st^ quartile: 3.01 ± 0.69, 4^th^ quartile: 2.39 ± 0.49, p < 0.001). This was mainly due to a gradual increase of resting velocities (1^st^ quartile = 26.3 ± 6.1 cm/s, 4^th^ quartile = 30.2 ± 6.4 cm/s, p < 0.001) while the reduction of hyperemic velocities remained unaffected (1^st^ quartile = 77.7 ± 18.9 cm/s, 4^th^ quartile = 70.9 ± 18.4 cm/s, NS). When age quartiles and ARFs were entered into a regression model, third and fourth age quartile (p < 0.0005 and p < 0.0001 respectively), left ventricular mass index (p < 0.0001), diastolic blood pressure (p < 0.001), total cholesterol (p < 0.002), fasting blood glucose (p < 0.01) and male gender (p < 0.05) were independent determinants of CFR in the whole population. Aging reduces coronary flow reserve in patients with angiographically normal coronary arteries due to a gradual increase of resting coronary flow velocity. CFR is also affected by atherosclerotic risk factors and left ventricular hypertrophy.

## Introduction

Coronary flow reserve (CFR) represents the maximal increase in coronary flow above its resting level for a given perfusion pressure when coronary vasculature is maximally dilated [1,2]. Conceptually, CFR is the difference between the basal, autoregulated coronary flow and the maximal flow, at any given perfusion pressure [3,4]. In the clinical setting, however, CFR is measured in dimensionless units by dividing maximal by autoregulated flow, that is the CFR ratio.

CFR can be measured by several - invasive or non invasive - techniques, which quantify coronary blood flow in absolute terms (e.g., positron emission tomography [PET]) or measure coronary blood flow velocity (Doppler) and calculate coronary flow velocity reserve [5]. Among these techniques, transthoracic echocardiography (TTE) allows the recording of flow velocities with a high feasibility for the mid-distal left anterior descending artery (LAD) [6]. TTE derived CFR of LAD has an excellent concordance with invasive Doppler flow wire and optimal reproducibility [6,7] and has now entered the stress echo laboratory for its clinical routine use during vasodilator stress testing.

A reduction in coronary flow reserve can be associated to a significant epicardial coronary artery stenosis, but also to coronary microvascular disease or to factors increasing extravascular resistance and endoluminal compressive forces with normal coronary arteries, as it happens in left ventricular (LV) hypertrophy, dilated or hypertrophic cardiomyopathy, aortic valve stenosis. Previous studies have suggested that CFR may be impaired in individuals with atherosclerotic risk factors (ARFs). Also aging induces similar effects and it is likely that CFR might be attenuated in elderly subjects free of coronary artery stenosis. An age-dependent reduction of CFR has been previously reported by PET in limited sample size of healthy volunteers [8,9]. The reduction of CFR can reflect a reduction in maximal flow with stable resting flow, stable maximal flow with increase in resting flow or a combination of reduction in maximal and increase in resting flow [1]. The primary end-point of the present study was to elucidate the most likely mechanism for the known decrease in CFR with age. Our hypothesis was that age and composite risk factors had differential impact on resting versus maximal coronary flow velocity.

## Methods

### Study population

From the EPIC (Echo Persantine International Cooperative Study) databank between August 2003 and June 2008, 694 patients were selected according to the following criteria: history of chest discomfort, dipyridamole echocardiography test (0.84 mg/Kg over 6’) (DET) performed before (within 15 days) coronary angiography; coronary angiography showing the absence of any degree of coronary artery stenosis in any major vessel or secondary branch. Of this initial population, 349 patients were excluded for previous revascularization procedures, resting or stress-induced abnormalities or inadequate echocardiographic image quality during stress precluding satisfactory imaging of LAD flow. After exclusions, 345 patients (134 women, age = 61.1 ± 13.3 years) represented the final study group*.* The indications for coronary angiography were chest discomfort refractory to therapy (n = 168), abnormal exercise electrocardiogram (n = 116) or abnormal perfusion scintigraphy (n = 61). Stress echocardiographic data were collected and analysed by stress echocardiographers not involved in the patient care. The diagnosis of arterial hypertension was defined in accordance with current European Society of Hypertension - European Society of Cardiology recommendations [10] and/or the need for antihypertensive drugs. Diagnosis of diabetes mellitus was based on the American Diabetic Association guidelines [11]. Hypercholesterolemia was defined according to the criteria of the European guidelines on cardiovascular disease prevention in clinical practice [12]. Cigarette smoking habit was self-reported. Data of anti-hypertensive and/or anti-ischemic therapy, if any, at time of testing were collected for each subject.

Subjects gave written informed consent and the study was approved by the Institutional Ethic Committee.

### Procedures

Twelve-hour fasting blood samples were obtained the same day as standard echocardiographic examination and CFR assessment. The measurements of lipid and glucose levels were performed by enzymatic methods (Boehringer Mannheim).

All the resting and stress echocardiographic examinations were done with commercially available ultrasound machines (Sequoia C256 Acuson Siemens, Mountain View, CA; Sonos 5500–7500 Philips Ultrasound, Andover MA; Vivid System 7, GE, Horten, USA) equipped with a miltifrequency phased-array sector scan probe and with harmonic technology. Echocardiographic examination at rest was recorded in order to obtain left ventricular (LV) quantitative analysis. LV end-diastolic and end-systolic volumes were measured by 2-D biplane method (modified Simpson’s rule) and LV ejection fraction (%) derived [13]. LV mass was calculated according to the recommended ASE formula and indexed for body surface area (g/m^2^) [13]. LV hypertrophy was defined as a LV mass >95 g/m ^2^ in women and > 115 g/m^2^ in men [13]. Besides the standard views for stress echo testing, specific views for LAD coronary artery was integrated into the cardiac imaging sequence. 2-D echocardiography and 12-lead ECG monitoring were performed in combination with high-dose dipyridamole (up to 0.84 mg over 6 min), in accordance to well established protocols [14,15]. During the procedure, BP and ECG were recorded each minute. Coronary flow in the mid-distal portion of LAD was searched in the low parasternal long-axis cross section under the guidance of colour Doppler flow mapping and attention was taken to maintain a constant incident (theta) angle (< 30°) between coronary flow and the Doppler beam during the entire duration of the tests [16]. All studies were digitally stored to simplify off-line reviewing and measurements. Coronary flow velocity parameters were analysed off-line by use of the built-in calculation package of the ultrasound unit. Flow velocities were measured at least twice for each study: at rest and at peak stress (before aminophylline injection). At each time point, three optimal profiles of peak diastolic Doppler flow velocities were measured, and the results were averaged. Coronary blood flow velocity reserve was defined as the ratio between hyperemic and resting peak diastolic coronary flow velocities Quality control of the diagnostic performance in the different centers was performed as previously described [17]. The intra- and inter-observer variability for measurements of Doppler-derived coronary flow assessment were <10% [18].

Coronary angiography in multiple views was performed according to the standard Judkins or Sones technique. At least five views (including two orthogonal views) were acquired for the left and at least two orthogonal views for the right coronary arteries, respectively. Additional appropriate projections were obtained in case of superimposition of side branches or foreshortening of the segment of interest. Two independent, blinded observers reviewed each angiogram. In case of disagreement (10% of cases), a third observer reviewed the exams and the judgment was binding.

### Statistical analysis

Statistical analyses were performed by SPSS package, release 12 (SPSS Inc, Chicago, Illinois, USA) Data are presented as mean value ± SD. Descriptive statistics was obtained by one-factor ANOVA and χ2 distribution with computation of exact p value by Monte Carlo method. Least squares linear regression was used to evaluate univariate and multivariate correlates of CFR measurements. For multiple linear regression model, multicollinearity was also examined by computation of in-model tolerance. The null hypothesis was rejected for p value < 0.05.

## Results

The study population was divided into quartiles of age: 1^st^ quartile = < 55 years, 2^nd^ quartile = ≥ 55 and 62 years, 3^rd^ quartile = between ≥ 63 years and 69 years, 4^th^ quartile = ≥ 70 years. Table 1 reports the clinical characteristics and main echocardiographic parameters according to age quartiles. As expected, body mass index and BP increased significantly with age. Heart rate was not significantly different among the various age groups. LV mass index significantly increasedwith aging. The prevalence of LV hypertrophy in the pooled population was 44% (150/345). The frequency of anti-hypertensive and/or anti-ischemic therapy at time of testing (not discontinued) was significantly higher in the 4^th^ quartile (p < 0.0001). Table 2 reports the prevalence of the main ARFs for age quartiles. Arterial hypertension had the greatest prevalence in the pooled population (54.8%), followed by hypercholesterolemia (46.7%), smoking habit (24.3%) and diabetes (20.3%) The frequency of arterial hypertension, hypercholesterolemia and, with a lower extent, diabetes mellitus increased with aging.

**Table 1:** Demographics and main echo characteristics of the study population according to age quartiles

	Age < 55 years	Age 55–62 years	Age 63–69 years	Age ≥70 years	p
	n = 85	n = 87	n = 85	n = 88	
Sex (M/F)	53 / 32	53 / 34	52 / 33	53 / 35	NS
Age (years)	42.7 ± 9.4	58.9 ± 2.2	66.4 ± 2.2	75.9 ± 4.4	<0.0001
	(40.6-44.7)	(58.5-59.4)	(65.9-66.9)	(74.9-76.8)	
BMI (Kg/m^2^)	25.3 ± 3.6	26.9 ± 3.9	26.8 ± 3.8	26.2 ± 3.9	<0.05
	(24.5-26.1)	(26.1-27.8)	(26.0-27.6)	(25.4-27.0)	
SBP (mmHg)	131.8 ± 15.7	139.3 ± 16.9	142.8 ± 18.3	146.5 ± 19.1	<0.0001
	(128.4-135.2)	(135.7-142,9)	(138.8-146.7)	(142.5-150.6)	
DBP (mmHg)	76.9 ± 10.4	81.1 ± 10.6	84.6 ± 9.9	86.1 ± 10.8	<0.0001
	(74.7-79.2)	(78.9-83.4)	(82.4-88.7)	(83.8-88.4)	
MBP ( mmHg)	95.3 ± 11.2	100.5 ± 11.6	103.9 ± 11.6	106.2 ± 11.9	<0.0001
	(92.8-97.7)	(98.1-103.0)	(101.5-106.5)	(103.7-108.7)	
HR (bpm)	69.9 ± 8.0	68.6 ± 8.8	71.1 ± 8.6	68.3 ± 8.2	NS
	(68.2-71.6)	(66.7-70.5)	(69.2-72.9)	(66.4-70.2)	
Total cholesterol (mg/dL)	174.3 ± 27.2	199.8 ± 43.7	202.1 ± 36.6	204.6 ± 41.3	<0.0001
	(168.4-180.2)	(190.5-209.1)	(192.2-208.1)	(195.9-213.4)	
Fasting blood glucose (mg/dL)	98.0 ± 19.9	102.4 ± 25.9	101.5 ± 24.9	99.5 ± 22.1	NS
	(93.7-102.3)	(96.9-107.9)	(96.1-106.9)	(94.8-104.1)	
EF (%)	60.4 ± 6.0	61.6 ± 5.6	60.8 ± 6.3	60.6 ± 5.6	NS
	(59.1-61.7)	(60.4-62.7)	(59.4-62.2)	(59.4-61.8)	
LVMi (g/m^2^)	97.7 ± 23.6	112.4 ± 28.7	110.3 ± 22.0	115.8 ± 24.7	<0.0001
	(92.6-102.8)	(106.3-118.5)	(105.6-115.1)	(110.5-121.0)	
Cardiac therapy (%, n)	20.0% (17)	47.1% (41)	54.1% (46)	59.1% (52)	<0.0001

Data expressed as mean ± SD (95% confidence interval for mean) or as percentage.

BMI = Body mass index, DBP = Diastolic blood pressure, EF = Ejection fraction, HR = Heart rate, MBP = Mean blood pressure, SBP = Systolic BP, LVMi = Left ventricular mass index.

**Table 2:** Atherosclerotic risk factors of the study population according to age quartiles

Factor	Age < 55 years	Age 55–62 years	Age 63–69 years	Age ≥70 years	P
Cigarette smoking	22.3% (19/85)	25.3% (22/87)	24.7% (21/85)	25.0% (22/88)	NS
Arterial hypertension	24.7% (21/85)	63.2% (55/87)	64.7% (55/85)	65.9% (58/88)	<0.01
Diabetes mellitus	17.6% (15/85)	19.5% (17/87)	22.4% (19/85)	21.6% (19/88)	<0.05
Hypercholesterolemia	12.9% (11/85)	55.2% (48/87)	60.0% (51/85)	57.9% (51/88)	<0.01

Data are expressed as percentage (number of patients with ARF / total number for age quartile).

Table 3 summarizes the data of the CFR test according to age quartiles. Mean CFR was 2.63 ± 0.71. In the whole population CFR was gradually reduced with increasing age, showing the lowest value in the fourth quartile of age (p < 0.0001). This reduction was accounted for an increase of resting coronary flow velocities while the hyperemic coronary flow velocities remained unaffected in the several age classes (p = 0.08, NS).

**Table 3:** Coronary flow reserve test according to age quartiles

	Age < 55 years	Age 55–62 years	Age 63–69 years	Age ≥ 70 yrs	p
CFR	3.01 ± 0.69 (2.87-3.16)	2.67 ± 0.54 (2.56-2.79)	2.47 ± 0.54 (2.36-2.59)	2.39 ± 0.49 (2.28-2.49)	<0.0001
CFV at rest (cm/s)	26.3 ± 6.1 (25.0-27.6)	28.7 ± 8.9 (26.8-30.7)	30.8 ± 8.8 (28.8-32.9)	30.2 ± 6.4 (28.6-31.5)	<0.001
CFV after Dip (cm/s)	77.7 ± 18.9 (73.5-82.0)	75.4 ± 22.9 (70.6-80.1)	74.6 ± 21.4 (69.9-79.1)	70.9 ± 18.4 (66.9-74.6)	NS

Data expressed as mean ± SD (95% confidence interval for mean).

CFR = Coronary flow reserve, CFV = Coronary flow velocity.

We also analysed the data excluding the 116 patients with objective signs of ischemia at study entry (by ECG criteria and/or perfusion abnormalities) in order to rule out microvascular angina as a potential confounder. In the subset of 219 patients with no objective signs of ischemia, CFR was also gradually reduced with increasing age (p < 0.001).

**Figure 1:**
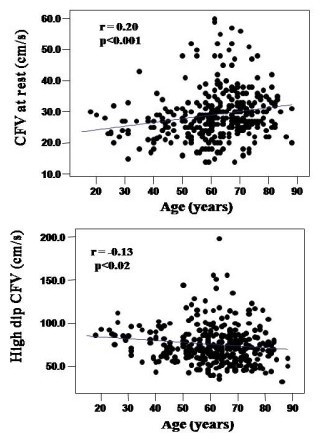
**Upper panel: Correlation between age and Doppler velocities at rest.** Lower panel: Correlation between age and Doppler velocities at hyperemic flow. Resting velocities progressively decrease with age, whereas hyperemic flow velocities remain unaffected.

**Figure 2:**
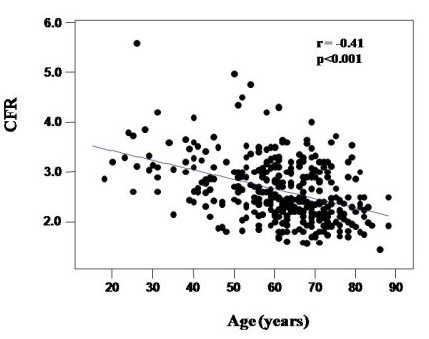
**Correlation between CFR and age.** CFR decreases progressively with aging.

In the pooled population age was correlated positively with coronary flow velocity at rest (r = 0.20, p < 0.0001) and negatively with hyperemic coronary flow velocity (r = −0.13, p < 0.02) (Figure 1). Age was negatively correlated with CFR (r = −0.41, p < 0.0001) (Figure 2) and was also related with systolic BP (r = 0.34), diastolic BP (r = 0.37) and mean BP (r = 0.39) (all p < 0.0001), with total blood cholesterol (r = 0.27) (p < 0.0001), fasting blood glucose (r = 0.18) (p < 0.001), body mass index (r = 0.12, p = 0.03) and LV mass index (r = 0.43, p < 0.0001).

A multiple linear regression analysis was performed to identify the independent associations of CFR, by including continuous variables (body mass index, heart rate, diastolic BP, total blood cholesterol, fasting blood glucose, LV mass index), categorical variables (male gender, cigarette smoking) and age quartiles as potential contributors (Table 4). CFR was independently associated with both the third (standardized β coefficient = −0.16, p < 0.005) and fourth (β = −0.22, p < 0.0001) age quartile. Among ARFs, elevated diastolic BP (p < 0.001) and high total cholesterol (p < 0.002) showed the best independent associations with CFR reduction, while the contribution of fasting blood glucose was lower (p < 0.01) and that of cigarette smoking did not achieve the statistical significance (p = 0.06). LVM index was a strong determinant of CFR (β = −0.32, p < 0.0001). Of note, by analyzing the impact of ARFs on hyperemic coronary flow velocity after adjusting for the same confounders analyzed in Table 4, only total blood cholesterol (standardized β coefficient = −0.126, diastolic BP (β = −0.134) and LV mass index (β = −0.130) (all p < 0.02) were independently associated with the hyperemic coronary flow velocity.

**Table 4:** Multiple independent correlates of CFR variables in the pooled population

	**Standardized Beta Coefficient**	**t**	**P**
Age quartile 2	−0.07	−2.01	0.201
Age quartile 3	−0.16	−2.77	<0.005
Age quartile 4	−0.22	−3.60	<0.0001
Male sex	−0.10	−2.08	<0.05
BMI	−0.03	−0.71	0.481
HR	−0.10	−2.23	<0.05
DBP	−0.17	−3.44	<0.001
Total cholesterol	−0.15	−3.06	<0.002
Fasting blood glucose	−0.12	−2.62	<0.01
Cigarette smoking	−0.08	−1.92	0.056
LVMi	−0.32	−6.66	<0.0001
Constant		15.44	<0.0001

Cumulative R^2^ = 0.350, SE = 0.50, p < 0.00001.

The age reference is the youngest quartile (18–54 years).

Abbreviations as in Table 1.

## Discussion

The present study demonstrates that the impact of aging on TTE-CFR in patients with angiographically normal coronary arteries is primarily due to the increase of coronary flow velocity at rest. Also ARFs and increased LV mass are associated with reduced CFR.

The influence of aging on CFR has been previously assessed using PET, in populations of limited size, which included healthy volunteers and not patients with ARFs [8,9]. In the first of these studies, by using 13 N-ammonia in 40 healthy volunteers, Czernin et al [8] demonstrated that aging did not alter significantly dipyridamole-induced myocardial hyperemic flow and that the gradual decline of myocardial blood flow reserve correlated with an age-related increase of myocardial work and blood flow at rest. In the other study, which used 16O-water PET on 56 normal volunteers, myocardial blood flow at rest and after hyperemia were roughly comparable up to 60 years of age, there was a significant increase in resting flow above 60 years and a significant reduction in hyperemic flow only above 70 years [9].

The present study extends these findings and is the first to analyze the impact of aging on CFR derived by TTE, in a multicenter large population of patients with ARFs in which the presence of any degree of coronary artery stenosis was excluded on the basis of coronary angiography. Aging reduces CFR and the impact of this impairment is increased as the number of ARFs increases. In this view, the mean values of CFR observed in each age quartiles cannot be assumed as normal reference values but reflect the cumulative impact of ARFs and of LV mass on coronary microcirculation, without the hemodynamic burden of coronary artery stenosis. The adverse influence of arterial hypertension, diabetes mellitus, hypercholesterolemia, cigarette smoking and LV hypertrophy on coronary microcirculation is well known [19-31]. It is worthy of note that in the present study total blood cholesterol, diastolic BP and LV mass index were independently associated to the reduction of hyperemic coronary velocity, true expression of impaired coronary microcirculation when epicardial coronary arteries are normal. Our results are also consistent with findings of Ahmari et al [32] who have demonstrated on 59 patients that traditional risk factors are related to a lower CFR in the face of a negative dobutamine stress echocardiography for wall motion criteria.. In the present study we evaluated intermediate risk patients with chest pain and/or positive test of inducible ischemia but totally normal coronary arteries at coronary angiography. In a recent study from our group [33], we assessed the prognostic impact of CFR in patients with normal or near-normal coronary arteries, identifying a subset of patients at higher risk of events when CFR was impaired. However, near–normal coronary arteries include subjects with subcritical lesions that may become troublemakers in the long-run, and at higher risk on the basis of coronary anatomy *per se* due to calcifications or soft plaques more prone to instability [34]. In this view, patients with near-normal coronary arteries were excluded from the present study, which included only 10 patients (3%) of the previous report [33].

### Study limitations

The study has some technical limitations corresponding to Doppler assessment of CFR. The main limitation is represented by the fact that coronary velocity ratio is a surrogate of flow reserve. Flow within the coronary artery cannot be calculated by transthoracic echocardiography because cross-sectional visualization of the vessel does not allow an accurate measurement of the diameter of the vessel. However, the estimated CFR can be considered accurate if the coronary functions only as a conduit, without changing in diameter during drug infusion. This assumption is reasonable by using vasodilating stressors such as adenosine and dipyridamole [35]. Another potential limitation corresponds to the determination of absolute coronary flow velocity which can be affected in some patients by the large incident (theta) angle between the blood flow velocity pattern and the Doppler beam. However, a < 30° angle theta was maintained constant for all the duration of the tests in the present study, without variation between resting and hyperemic conditions.

In conclusion, the age-related effect on coronary flow reserve is mainly due to the progressive increase of resting coronary flow velocity at rest. Coronary flow reserve is impaired also by the combined action of atherosclerotic risk factors and by the additional effect of increased LV mass. The present results have important clinical implications since demonstrate the cumulative effect of aging and traditional atherosclerotic risk factors on coronary flow reserve in the absence of demonstrable epicardial coronary artery disease. Aging is not modifiable but its effect should be taken into account when weighing its clinical role with other factors affecting coronary microvascular function, especially for the values of CFR ranging between 2 and 3. Standards of normality for CFR should be normalized for age, as it is the case for other echocardiographic parameters, such as wall thickness or Doppler-derived diastolic measurements and pulmonary arterial systolic pressure.

## Misc

Financial support for the present study was received from institutional funding of the CNR, Institute of Clinical Physiology, Pisa, Italy

## Competing interest

The authors declare that they have no competing interests.

## Authors contribution

MG conceived of the study and participated in its design and coordination, performed the statistical analysis and drafted the manuscript, FR, SG and LC participated in the study design and coordination and performed echo scans, CS participated in the study coordination and performed echo scans, RS and EP participated in the study design, performed and revised the statistical analysis and revised the manuscript. All authors read and approved the final manuscript.
